# Twitter-Based Influenza Detection After Flu Peak via Tweets With Indirect Information: Text Mining Study

**DOI:** 10.2196/publichealth.8627

**Published:** 2018-09-25

**Authors:** Shoko Wakamiya, Yukiko Kawai, Eiji Aramaki

**Affiliations:** 1 Nara Institute of Science and Technology Ikoma Japan; 2 Kyoto Sangyo University Kyoto Japan; 3 Osaka University Osaka Japan

**Keywords:** influenza surveillance, location mention, Twitter, social network, spatial analysis, internet, microblog, infodemiology, infoveillance

## Abstract

**Background:**

The recent rise in popularity and scale of social networking services (SNSs) has resulted in an increasing need for SNS-based information extraction systems. A popular application of SNS data is health surveillance for predicting an outbreak of epidemics by detecting diseases from text messages posted on SNS platforms. Such applications share the following logic: they incorporate SNS users as social sensors. These social sensor–based approaches also share a common problem: SNS-based surveillance are much more reliable if sufficient numbers of users are active, and small or inactive populations produce inconsistent results.

**Objective:**

This study proposes a novel approach to estimate the trend of patient numbers using indirect information covering both urban areas and rural areas within the posts.

**Methods:**

We presented a TRAP model by embedding both direct information and indirect information. A collection of tweets spanning 3 years (7 million influenza-related tweets in Japanese) was used to evaluate the model. Both direct information and indirect information that mention other places were used. As indirect information is less reliable (too noisy or too old) than direct information, the indirect information data were not used directly and were considered as inhibiting direct information. For example, when indirect information appeared often, it was considered as signifying that everyone already had a known disease, leading to a small amount of direct information.

**Results:**

The estimation performance of our approach was evaluated using the correlation coefficient between the number of influenza cases as the gold standard values and the estimated values by the proposed models. The results revealed that the baseline model (BASELINE+NLP) shows .36 and that the proposed model (TRAP+NLP) improved the accuracy (.70, +.34 points).

**Conclusions:**

The proposed approach by which the indirect information inhibits direct information exhibited improved estimation performance not only in rural cities but also in urban cities, which demonstrated the effectiveness of the proposed method consisting of a TRAP model and natural language processing (NLP) classification.

## Introduction

### Background

The increased use of social networking platforms entails more widely shared personal information. Twitter, a microblogging platform that enables users to communicate by updating their status using 140 or fewer characters, has attracted the attention of many researchers and service developers as a valuable personal information resource. Consequently, various approaches for analyzing social data (called as *social monitoring* [1]) have been presented so far. These approaches have presented an important shared premise that Twitter users can be human sensors for event detection [2], and the feasibility of these approaches has been demonstrated on various occasions such as earthquakes [2-4], political elections [5-7], stock market fluctuations [8], and outbreaks of various infectious diseases [9-33]. Among them, the study of social monitoring of health-related information shared on the internet is referred to as *infodemiology* [1,34] and gathers much attention in terms of practical needs.

### Objective

**Figure 1:**
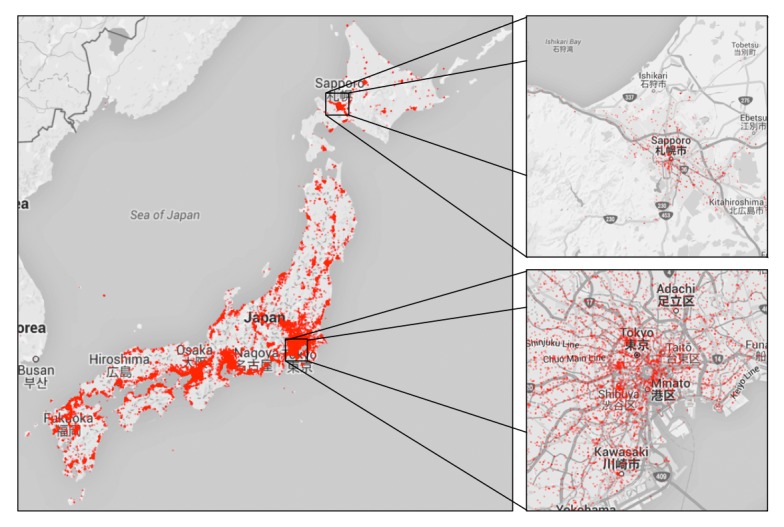
Population bias in Twitter-based influenza surveillance. According to the geographic distribution in Japan of 7,666,201 influenza-related tweets for the period from 2012 to 2015, most Twitter users are in urban cities (such as Tokyo and Osaka). Other cities are adversely affected by a shortage of data that biases influenza detection there.

This study particularly examined such applications for detecting disease epidemics, by taking advantage of the swiftness of the information transmission on Twitter. Numerous Twitter-based disease detection and prediction systems have been developed worldwide. However, these systems have several weaknesses. One significant deficit is population distribution imbalance owing to the fact that most social networking service (SNS) users reside in urban areas, resulting in analysts facing difficulty getting sufficient amounts of data from rural areas. For example, user population of Japan is strongly concentrated in a few central cities such as Tokyo and Osaka. Specifically, the population of Tokyo is estimated to be 13.515 million (about 11% of Japan’s total population) [35]. Other users live outside these areas, in less populated regions of Japan. This population bias results in difficulties in obtaining consistent performance. Figure 1 shows the geographic distribution in Japan of 7,666,201 influenza-related tweets for the period from 2012 to 2015. The distribution is skewed because rural areas have fewer young people than the cities. For instance, the number of young in-migrants (aged 15-29 years) from other areas to Tokyo was 20.56% as of 2014. The other areas except Osaka and Nagoya basically suffer from an exodus of young people [36]. Therefore, fewer SNS users are available in the rural areas.

To overcome this skewed distribution problem, information from a broader range of targets than that used in earlier studies can be utilized. One solution is to use indirect information [37,38] that had been discarded in previous studies related to Twitter-based disease surveillance [15,26-31,39]. Examples of such indirect information are as follows:

1. *My friend in Hokkaido caught the flu.*
2. *NEWS: Classes in Hokkaido have been suspended because of the flu.*

**Figure 2:**
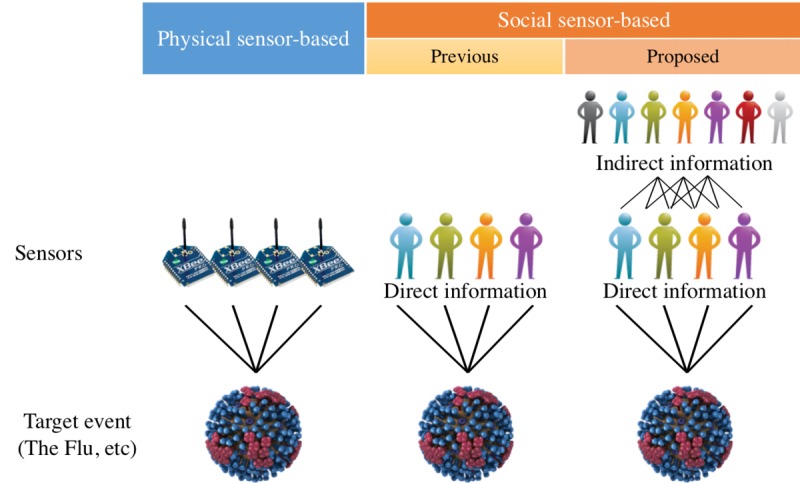
Most social sensor–based approaches consider people as sensors (center and right). Whereas previous social sensors exploited only direct information, the proposed method uses indirect information (right).

The fundamental idea is presented in Figure 2. Although tweets are concentrated in the urban areas, indirect information covers wider areas. However, indirect information is unreliable (sometimes too noisy or too old). In example (1) above, it is unknown when the *friend* caught the flu. And in example (2), the flu had already spread to the area. Due to the difficulties presented above, previous studies did not use such indirect information to any significant degree.

**Figure 3:**
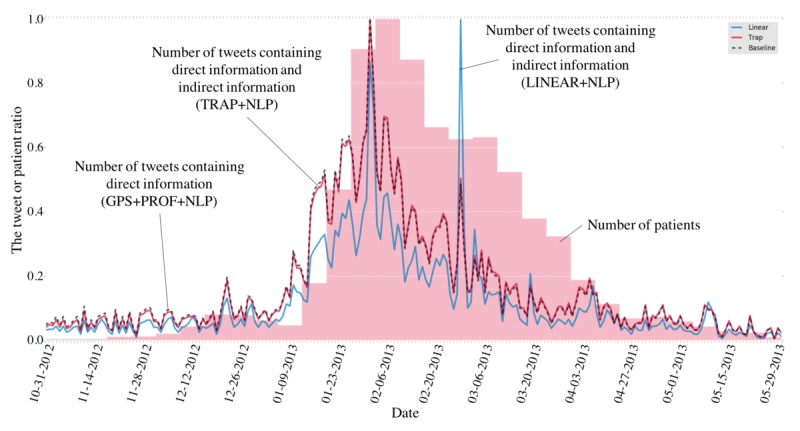
Amounts of direct and indirect information in a tweet timeline in Hiroshima from November 1, 2012 to May 31, 2013. The black dashed line shows the timeline of direct information (BASELINE+PROF+NLP), the blue line shows the timeline of direct information and indirect information that are aggregated in a naive way (LINEAR+NLP), the red line shows the timeline of direct information and indirect information that are aggregated by the proposed model (TRAP+NLP), and the red area shows gold standard timeline. The x-axis shows the date, and the y-axis indicates the tweet ratio and the patient ratio (normalized by the max value in the season). GPS: global positioning system; PROF: profile information; NLP: natural language processing.

An example of tweet timelines and a patient timeline is presented in Figure 3. Note that each timeline is normalized based on the maximum value of a season. Direct information (black dashed line) shows a similar timeline to the patient timeline (gold standard; red area). However, before the peak of epidemics, the amount of direct information increases a bit, leading to overestimation errors. In addition, after the peak of epidemics, the amount of direct information decreases, leading to underestimation errors. On the other hand, the timeline of the linear combination of direct and indirect information (blue line) shows complex phenomena: it has many and sometimes sudden peaks (eg, February 27, 2013), which would be caused by news spreading and so on. Apparently, indirect information is difficult to use.

To aggregate direct information and indirect information in a sophisticated way, this study employed a different approach that specifically examines the relation between indirect information and the human motivation to tweet. The approach considers that after the peak of epidemics, the topic of influenza goes out of fashion, inhibiting the motivation of people to tweet about the flu. Consequently, a more similar timeline (red line) to the patient timeline (gold standard; red area) than that of the direct information timeline can be obtained as shown in Figure 3. It also could screen out sudden peaks of the amount of indirect information.

Another difficulty is the detection of the degree of the propagated information. This study specifically examines the amount of indirect information because it indicates that people in different places also know about the event. Consequently, this study made the following assumption: the degree of propagation (popularity) is correlated with the amount of indirect information. According to the previous study by Aramaki et al [15], most people report influenza information precisely in the early stage of an influenza season. However, as the indirect information is propagated widely, most people know about the influenza epidemic and become insensitive to the event. We designate such deactivated people as trapped sensors. This study investigates the degree to which this model improves the performance of the event detection.

The objective of this study was to handle indirect information to estimate the trend of the number of influenza patients in each area and each season. This estimation would be useful in satisfying practical needs not only in the industry but also of individual consumers, such as the supply control of vaccines and products for disease prevention or treatment. To study this, we built a state-of-the-art Twitter-based influenza surveillance system. Our contributions are 2-fold:

1. We reconfirmed the contribution of existing techniques. The existing techniques mainly consist of 2 main parts: tweet classification based on natural language processing (NLP) techniques and the use of direct information comprising global positioning system (GPS) information and profile information (PROF).
2. Subsequently, we evaluated the proposed model that aggregates indirect information to direct information.

Although a Twitter platform based on the Japanese language is used in this study, the proposed model for aggregating social sensors is universal, as they do not depend on a specific platform or language because no platform and language-specific technique are used. Note that the proposed model does not always work better under all conditions; we at least showed that our results targeted larger number of areas (47 areas) compared with previous studies to achieve a higher accuracy on an average.

## Methods

### System Overview

The system consisted of 3 modules to analyze given tweet data: a positive or negative (P or N) classification module, a location detection module, and a data aggregation module. For the aggregation, we used 2 methods using 3 types of location information: a LINEAR model and a TRAP model.

### Tweet Data Collection

We collected the influenza-related tweets written in Japanese via the Twitter streaming application programming interface (API) for 5 years (from August 2, 2012 to March 1, 2016). All tweets comprised an influenza-related Japanese keyword *I-N-FU-RU* (*flu* in Japanese). These data include noise tweets, which are tweets that do not index an influenza patient. An example of such noise tweets is *influenza vaccination*. To filter out such influenza-negative tweets, the NLP module determines whether a given tweet is positive or negative.

### Natural Language Processing Module: Positive or Negative

This module judges whether a given tweet is of an influenza patient (positive) or not (negative). This task is a sentence binary classification such as spam email filtering. This module applied a binary classification based on support vector machine under the bag-of-words representation. In the implementation, the same classification model was used as in the study by Aramaki et al [15]. To construct the model, 5000 tweets as a training set were assigned one of the two labels: positive or negative (P or N) by human annotators. In this labeling, tweets that met the following 2 conditions are regarded as a positive case:

- *Condition 1:* Area—Although a tweet seems to report a positive case, it may be not about a Twitter user himself or herself but about others. In such a case, we assume that one or more people with influenza would be likely to be present around the Twitter user. Here, we regard *around* as a distance in the same city. For cases in which the distance is unknown, we regard it as negative. Due to this annotation policy, the retweet type message is also negative.
- *Condition 2:* Tense—The tense should be present tense (current) or recent past. Here, we define the *recent past* as the prior 1-day period: *the previous day.*

The training set consisted of pairs of sentences and a label (positive or negative). Samples of tweets with labels are shown as follows:

1. BBC News: Okinawa has an influenza pandemic—(P, I)
2. Okinawa suffers a major outbreak of influenza—(P, D)
3. Retweet: My mother got the flu today—(P, I)
4. I got an influenza shot today—(N, D)
5. Doctor said influenza will be late in this season—(N, I)

Note that P/N denotes positive (P) or negative (N); D/I denotes Direct information (D) or Indirect information (I). We use retweet, too, in the same manner as normal tweets (non-retweet tweets).

For classifying a test set of tweets, we split each Japanese sentence into a sequence of words using a Japanese morphological analyzer MeCab (ver.0.98) [40] with IPADic (ver.2.7.0) [41]. The parameters for support vector machine including a polynomial kernel (d=2) were used in the study by Aramaki et al [15].

### Location Detection Module (Direct or Indirect)

We used 3 types of location information extracted from each tweet: direct information, which includes GPS information and profile information, and indirect information or referred location.

#### Direct Information: Global Positioning System (GPS) Information

A tweet contains GPS-based data if a Twitter user allows the use of the location function. However, most users turn this functionality off for privacy reasons. Currently, the ratio of tweets with GPS information is only 0.46% (35,635/7,666,201) in our dataset.

#### Direct Information: Profile Information (PROF)

Several Twitter users describe their address in their profile (PROF). We regard the Twitter user as near the profile address. The proportion of tweets with profile location is 26.23% (2,010,605/7,666,201). This information was used in the study by Aramaki et al [15]. To disambiguate the location names, we used a geocoding service [42] provided by Google Maps [43]. Specifically, we sent queries about Twitter users’ locale to Google Maps and obtained results in JavaScript Object Notation format. We wrote a simple parser in Python to parse these returned results to get information about the country.

#### Indirect Information: Referred Location

Several tweets contain the location name in the contents, such as “My friend in Hokkaido caught the flu.” This study used this indirect information. To detect the location name in the contents, we used a location name list consisting of area names and famous landmarks. The proportion of tweets with indirect information was 4.73% (362,349/7,666,201).

Thus, we use the location if the GPS information is available. Otherwise, if a user profile information includes address data, then we use that information. The address data are geocoded by the geocoding service, API, provided by Google. Otherwise, if the content of the tweet contains a location name (area names), we consider it as the indirect information in the area. Consequently, a tweet is classified into GPS, PROF, or indirect information. Note that this classification is partly inclusive, as a tweet is classified into GPS or PROF exclusively, and then the tweet including location name is also counted as indirect information inclusively.

#### Aggregation Module (LINEAR or TRAP)

A difficulty hindering the combination of different resources is the question of how to combine them. This study investigated 2 methods: (1) simple aggregation (LINEAR model) and (2) TRAP model, which is proposed for implementing our assumption that people prefer to report new information and that they are insensitive to already-propagated information.

##### LINEAR Model

A simple method to use indirect information is to aggregate different types of information. In this model, we weigh the direct information as more important than the indirect information.

We formalize the number of patients *I*_LINEAR_*(a,t)* in area *a* at day *t* as follows:

*I_LINEAR_(a,t)* = *w_GPS_ · GPS(a,t)* + *w_PROF_ · PROF(a,t)* + *w_IND_∑_b∈A_ IND(a,b,t)* (**1**)

Where, *GPS(a,t)* is the number of tweets with GPS information, *PROF(a,t)* is the number of tweets with profile information, *IND(a,b,t)* is the number of tweets with indirect information, and *w_GPS_*, *w_PROF_*, and *w_IND_* are weight parameters.

##### TRAP Model

This model includes the following 2 assumptions:

1. People prefer a new event and are, therefore, insensitive to an already-propagated event.
2. The degree of propagation (popularity) is correlated with the amount of indirect information.

The first assumption derives from human nature—people hesitate to inform others of an already-known fact. For example, if the Twitter stream is full of repeated influenza information, then such a situation dampens enthusiasm to tweet similar information.

The second assumption comes from the features of Twitter. Most indirect information consists of retweet or news information that tends to delay the direct information. The volume of this type of information corresponds to the volume of people who never tweet.

**Figure 4:**
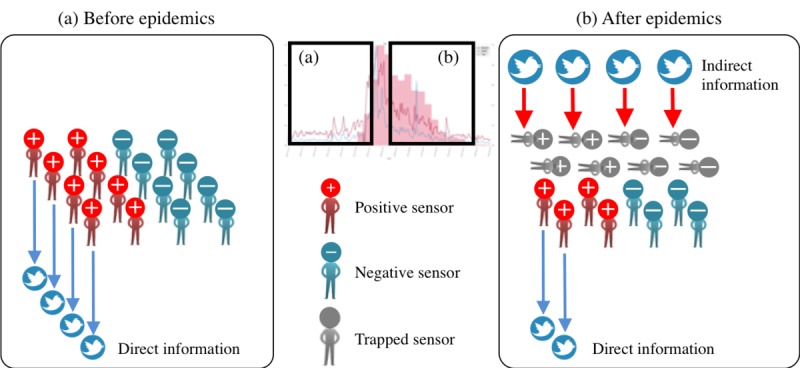
Concept image of TRAP model. (a) People actively report the influenza before epidemics. (b) However, most people lose interest in sharing the direct information after epidemics because much indirect information already exists. In the proposed model, we call such people Trapped Sensors.

On the basis of these 2 assumptions, in the early stage of a season, most social sensors are *activated* to report influenza precisely (see Figure 4). Because the indirect information spreads widely, most people become *deactivated* to the event (Figure 4). We designated such deactivated people as trapped sensors. Under these circumstances, although the number of influenza tweets is small, the number of patients might be larger than the tweet volume, because a trapped sensor might disregard influenza.

We formalize the number of patients *I_TRAP_(a,t)* in area *a* at day *t* using a popularity function, *pop(a,t)*, as follows:

*I_TRAP_(a,t)*=*(I_LINEAR_[a,t])* / *(w_USERS_∙N_a_* – *w_TRAP_∙* log*(pop[a,t]* + *1)* (2)

*pop(a,t)*=*∑^t^_d=1_IND(a,d)*

Where *I_LINEAR_(a,t)* is the linear model and variable *N_a_* is a set based on the number of potential active tweeting users defined by the number of tweets. A function, *pop(a,t)*, returns a cumulative number of the indirect information by the day *t* in a season, indicating the degree of popularity of attention of a crowd to influenza in the area *a*. *w_USERS_* and *w_TRAP_* are weight parameters.

**Table 1:** Data description.

Season	Duration	Number of tweets (size)
SEASON 2012	November 1, 2012-May 31, 2013	1,959,610 (729.4 MB)
SEASON 2013	November 1, 2013-May 31, 2014	501,542 (143.7 MB)^a^
SEASON 2014	November 1, 2014-May 31, 2015	2,736,685 (808.2 MB)
ALL	August 2, 2012-March 1, 2016	7,666,201 (2.275 GB)

^a^We were unable to collect sufficient tweets on January 17, 2014 and January 18, 2014 in SEASON 2013 because of Twitter application programming interface specification changes. In addition, the number of tweets throughout this season was consistently smaller than the other seasons.

### Evaluation

#### Datasets

These results were obtained by using the Japanese infectious disease data consisting of 2 types of data: one is Twitter data for the proposed system, and the other is the timeline report of the number of influenza patients.

#### Tweet Data

Our data comprised a collection of influenza-related tweets spanning 5 years. Human annotators annotated the collected tweet data into positive or negative labels, and using the support vector machine-based classification model constructed in the previous work [15] trained with a sample of 5000 randomly selected tweets from an influenza tweet corpus from November 2008, we classified our collected data into positive or negative label. For more precise information regarding the classifier and the training set, please see the previous report by Aramaki et al [15].

Because influenza epidemics appear in the winter, we split the data as follows:

1. SEASON 2012: November 01, 2012 to May 31, 2013
2. SEASON 2013: November 01, 2013 to May 31, 2014
3. SEASON 2014: November 01, 2014 to May 31, 2015

Statistics of the tweet data are presented in Table 1. Note that we were unable to collect sufficient tweets in SEASON 2013 because of changes in Twitter API specification, and we only used what we collected.

#### Gold Standard Data

We used the number of influenza cases as the gold standard data. In Japan, the Infectious Disease Surveillance Center [44] gathers statistics of patients diagnosed with influenza by rapid influenza diagnostic tests from about 5000 clinics and releases summary reports called the Infectious Diseases Weekly Reports [45]. The report presents the number of influenza patients for each Japanese prefecture (47 areas) in a week. Therefore, this test set enables week-based evaluation in 47 areas.

### Models

We compared the 4 methods described below.

#### TRAP

TRAP is the proposed model. It detects disease epidemics by considering the balance between direct information (GPS information and profile information) and indirect information (referred location). In this study, we set *N*_a_ to a value based on the number of potential active tweeting users for equation 2. Afterward, we set the weight parameters *w_USERS_* and *w_TRAP_* to 0.1 and 2.0, respectively, based on the results of preliminary experiments.

#### LINEAR

LINEAR is a model that uses GPS information, profile information, and indirect location information together. In this study, weight parameters *w_GPS_*, *w_PROF_*, and *w_IND_* in equation 1 were set to 1.0. Note that these values are not optimal parameters. This study set the weighting parameters based on heuristic and preliminary experimental results. To examine optimal parameters for improving the validity of our model is one of the future works.

#### BASELINE+PROF

This is a baseline model presented in the study by Aramaki et al [15]. The approach uses GPS information and profile location:

*I_BASE + PROF_(a,t)*=*GPS(a,t)* + *PROF(a,t)* (3)

#### BASELINE

This is a simple baseline that uses only GPS information:

*I_BASE_(a,t)*=*GPS(a,t)* (4)

In addition to evaluation of the effectiveness of the positive or negative classification (NLP technique), we also conducted with or without the test. Thus, with the various combinations, 8 methods (4×2) were evaluated (see Multimedia Appendix 1).

### Evaluation Metric

The evaluation metric used in this study is the correlation (Pearson correlation coefficient) between the gold standard values and the estimated values. This metric is also used in the previous study [33]. The correlation-based evaluation is unbiased under the assumption of equal population sizes. Therefore, we can calculate the correlation coefficient, *r*, for a given data array consisting of the gold standard data (the number of patients) and the values that a model estimated based on the number of tweets.

We regard strong positive correlation as high performance, which comes from the previous studies [15,33]. Specifically, we defined a strong positive correlation as *r*>.7, moderate positive correlation as .4<*r* ≤.7, and weak positive correlation as 0<*r* ≤.4.

## Results

### Overview

Evaluation was performed for 4 durations: (1) SEASON 2012, (2) SEASON 2013, (3) SEASON 2014, and (4) SEASON-TOTAL (all; 1-3). Thus, 1504 (8 methods×47 areas×4 durations) correlation coefficients were calculated.

Table 2 presents the results obtained. Table 2 and Table 3, respectively, present the correlation coefficients of models with and without NLP for the gold standard data. Note that most of the correlation coefficients (99.60%,1498/1504) were positive, and a high negative correlation was not observed. Specifically, we discuss these results in terms of contributions of NLP-based classification, profile information, and data aggregation by LINEAR model and TRAP model.

**Table 2:** Values of the correlation coefficient (*r*) of methods with natural language processing.

Target and method		SEASON 2012	SEASON 2013	SEASON 2014	SEASON-TOTAL
**All areas**					
	TRAP+NLP^a^	.76^b^	.70^b^	.69^b^	.70^b^
	LINEAR+NLP	.70	.55	.53	.50
	BASELINE+PROF^c^+NLP	.74^d^	.68	.67	.69
	BASELINE+NLP	.33	.37	.48	.36
**High-population areas (Top 10)**					
	TRAP+NLP	.80^b^	.77^b^	.72^b^	.75^b^
	LINEAR+NLP	.78^d^	.65	.64	.64
	BASELINE+PROF+NLP	.80^b^	.77^b^	.71^d^	.75^b^
	BASELINE+NLP	.55	.60	.63	.53
**Low-population areas (Top 10)**					
	TRAP+NLP	.75^b^	.66^b^	.71^b^	.69^b^
	LINEAR+NLP	.62	.46	.48	.43
	BASELINE+PROF+NLP	.70	.61	.65	.64
	BASELINE+NLP	.21	.26	.35	.25

^a^NLP: natural language processing.

^b^Highest correlation coefficient in each target area and each SEASON.

^c^PROF: profile information.

^d^High correlation (*r*>.7).

**Table 3:** Values of correlation coefficient (*r*) of methods without natural language processing.

Target and method		SEASON 2012	SEASON 2013	SEASON 2014	SEASON-TOTAL
**All areas**	
	TRAP	.72^a^	.63^a^	.67^a^	.65^a^
	LINEAR	.65	.48	.53	.48
	BASELINE+PROF^b^	.69	.59	.66	.64
	BASELINE	.29	.34	.48	.35
**High-population areas (top 10)**	
	TRAP	.75^a^	.69^a^	.71^a^	.70^a^
	LINEAR	.72^c^	.60	.63	.61
	BASELINE+PROF	.75^a^	.69^a^	.70	.70^a^
	BASELINE	.44	.56	.63	.50
**Low-population areas (top 10)**	
	TRAP	.71^a^	.61^a^	.64	.60^a^
	LINEAR	.58	.41	.46	.40
	BASELINE+PROF	.65	.52	.65^a^	.59
	BASELINE	.20	.23	.35	.25

^a^Highest correlation coefficient in each target area and each SEASON.

^b^PROF: profile information.

^c^High correlation (*r*>.7).

### Contribution of Natural Language Processing–Based Classification (TRAP Vs TRAP+NLP)

To evaluate the contribution of NLP for positive and negative classification, we compared the results of TRAP in Table 3 and TRAP+NLP in Table 2. Although both methods are strongly correlated with the gold standard data, TRAP+NLP (*r*=.70 in SEASON-TOTAL) is predominantly higher than TRAP (*r*=.65). This result demonstrates the contribution of NLP.

In addition, TRAP+NLP and all other models with NLP (BASELINE+NLP, BASELINE+PROF+NLP, and LINEAR+ NLP) achieved better detection performance using the NLP classifier.

Although methods with NLP worked well to estimate influenza epidemics, almost half of the tweets were removed. This might indicate that the NLP-based classification used in this domain (influenza or not) is basically simple, so it must be improved.

### Contribution of Profile Information (BASELINE+NLP Vs BASELINE+PROF+NLP)

To evaluate the contribution of profile information, we compared BASELINE+NLP with BASELINE+PROF+NLP. As shown in Table 2, the correlation coefficient of BASELINE+ PROF+NLP (*r*=.69 in SEASON-TOTAL) is much higher than that of BASELINE+NLP (*r*=.36) through all SEASONs. This fact suggests that the profile information is highly related to improving the performance in detecting influenza epidemics. However, BASELINE+NLP achieved lower correlation in this study than in Aramaki et al [15]. One of the possible reasons would be that the model did not consider an area (prefecture)-level estimation, so it did not work well in several areas that did not have enough number of tweets.

As described above, both NLP classification and profile information improved the performance to detect influenza epidemics. This result shows that the combination of these techniques (BASELINE+PROF+NLP) achieved higher performance.

### Contribution of Indirect Information in LINEAR Model (BASELINE+PROF+NLP Vs LINEAR+NLP)

To evaluate the contribution of indirect information in the LINEAR model, we compared the performance of BASELINE+PROF+NLP with LINEAR+NLP. Although the performance of both methods was medium, the correlation coefficient of LINEAR+NLP (*r*=.50 in SEASON-TOTAL) is lower than that of BASELINE+PROF+NLP (*r*=.69) through all SEASONs, as shown in Table 2. This point indicates the difficulty inherent in detecting influenza epidemics solely by adding indirect information in a naive manner.

### Contribution of Indirect Information in TRAP Model (BASELINE+PROF+NLP Vs TRAP+NLP)

To evaluate the proposed model, the TRAP model, we compared the respective performances of TRAP+NLP and BASELINE+ PROF+NLP, which were better than LINEAR+NLP.

In fact, TRAP+NLP exhibited the highest correlation coefficient among the models, indicating that it achieved the best performance for influenza epidemic detection on the gold standard data. This, in turn, suggests that TRAP model methods effectively contribute to the exploitation of both direct and indirect information from social sensors to detect disease epidemics accurately.

## Discussion

### Few Tweets After Flu Peak

The fact that the TRAP model outperforms the LINEAR model indicates that when influenza becomes a hot topic, people do not talk about it, which shows the aspect of human nature in which people become bored quickly with the news. Similar phenomena have also been presented from a psychological viewpoint. Most studies showed rapid propagation of rumors (especially bad news) and their short life [46-48]. Among various SNSs, Twitter is an extremely *fast* media. Therefore, the life of news on this platform might be shorter than other existing news. In other words, people might hesitate to tweet an already-known fact.

This model has sufficient room for application to additional studies. For example, we simply regard the simulation of the referred tweet as news. Better methods using other media, such as news website information, are reasonable. The manner of estimation of the potential tweet users can also be improved by considering more realistic data.

#### Effectiveness of Each Module

From results obtained from the experiment presented in the previous section, we observed the following 3 findings:

1. Effectiveness of NLP-based classification.
2. Effectiveness of direct information and indirect information.
3. Effectiveness of data aggregation by TRAP model.

We first reconfirmed the 2 findings that were already studied in the previous work [15]—the effectiveness to apply NLP-based tweet classification and the effectiveness to use direct information. Then, we evaluated the effectiveness to use indirect information, in addition to direct information and to embed this information into TRAP model that are the main contributions of this paper.

Another novelty of this study is high-resolution geographic analysis. Therefore, we discuss the above effectiveness for each area throughout this section. Multimedia Appendix 2 portrays temporal changes of the gold standard data (red bar plot) and results of TRAP+NLP (red line), LINEAR+NLP (gray line), and BASELINE+PROF+NLP (blue line) for 3 SEASONs in 47 areas in Japan. Note that our evaluation was conducted by comparing the correlations between a tweet timeline and a patient timeline in an area. We assumed that the comparison would not be biased if the population sizes were comparable.

#### Effectiveness of Natural Language Processing–Based Classification

We determined the effectiveness of NLP-based classification by comparing the performance of the methods with NLP for the top-10 high-population areas in Table 2 with the performance of the methods without NLP for the top-10 low-population areas in Table 3. The rank of the population of areas is presented in Multimedia Appendix 3.

In urban areas such as Tokyo and Osaka, the TRAP model (without NLP) performance was sufficiently high. In fact, the correlation coefficient of TRAP was equal to or higher than .7. For the other results, all correlation coefficient values were higher than .5, reflecting medium correlation.

However, in more rural areas such as Shimane and Toyama, no significant improvement was observed when NLP was used. In particular, little difference in performance was found between BASELINE+NLP and BASELINE. However, NLP never worsened the performance, which motivates the use of NLP.

#### Effectiveness of Profile Information and Propagated Information

The proposed method used 3 types of location information: GPS information, profile information (as used by previous studies), and referred location. We discussed the effects of exploiting the referred location (as indirect information), as well as GPS information and profile information (as direct information). From Table 2, we observed that the indirect information might not be as important in high-population areas such as Tokyo and Osaka. For example, BASELINE+PROF+NLP realized a high correlation (*r*>.7) in urban areas on an average. In such areas, even BASELINE+NLP only using GPS information had medium correlation.

In contrast, using indirect information was effective in rural areas. Although BASELINE+PROF+NLP was determined as just medium correlation (*r* ≤.7) through all SEASONs, TRAP+NLP showed high correlation in SEASON 2012 and SEASON 2014, as shown in Table 2. The results for SEASON 2013 might be affected by the lack of tweet data, as shown in Table 1.

This result might be caused by a common pattern by which much direct information is available in urban areas. In contrast, because a sufficient amount of direct information is not available from rural areas, there is some lack of exploitation of indirect information.

#### Effectiveness of Data Aggregation by TRAP Model

We can discuss the effectiveness of the TRAP model by comparing the correlation coefficients of the top-10 high-population areas and that in the top-10 low-population areas in Table 2.

In urban areas, the performance of 2 methods related to the TRAP model (TRAP+NLP and TRAP) was the highest among the others. The correlation coefficients of the 2 methods related to the LINEAR model (LINEAR+NLP and LINEAR) were less than .7, except in SEASON 2012. For example, for Tokyo (AREA13) and Osaka (AREA27) in Multimedia Appendix 2, TRAP+NLP matched the gold standard data well. In contrast, LINEAR+NLP has some gaps. These results confirm the effectiveness of TRAP model for tweets in urban areas.

In rural areas, the performance of the methods related to the TRAP model (TRAP+NLP and TRAP) was also the highest. Most of the correlation coefficients were higher than .6. In particular, the performance of TRAP+NLP in the rural areas was higher than that of the LINEAR+NLP in the urban areas on an average. For example, for Shimane (AREA32) and Toyama (AREA18) in Multimedia Appendix 2, the results of both TRAP+NLP and LINEAR+NLP in SEASON 2012 matched the gold standard well. However, the results in other SEASONs have partial gaps. The results of LINEAR+NLP are affected by the small number of tweets. For such areas, we improve the performance by adjusting the weight parameters adequately.

Overall, we confirmed the effectiveness of aggregation using the TRAP model that does not treat the 3 types of location information in the same manner but instead distinguishes referred location as indirect information and uses it differently.

### Relation Between Volume of Tweets and Performance

The relation between population and the detection performance presents an important finding. Multimedia Appendix 3 presents the relation between population (blue bar plot) of each area and performance (lines). The population is the number of tweets. The performance is the correlation coefficient. This figure compares TRAP+NLP (red line) with BASELINE+PROF+NLP (dotted black line).

The results show that the performance of TRAP+NLP was higher than that of BASELINE+PROF+NLP in urban areas. Specifically, the top 17 high-population areas (from Tokyo [AREA13) to Ibaraki [AREA8]) exhibited high correlation (*r*>.7). In these areas, more than 400 tweets were emitted.

However, other areas have large performance variances. Although both methods sometimes stagnate at the same performance level, in most cases, TRAP+NLP outperforms BASELINE+PROF+NLP. In Aomori (AREA2), Nagano (AREA17), Oita (AREA44), Nagasaki (AREA42), and Yamanashi (AREA16), the TRAP model achieved higher performance (*r*>.7) than that of the BASELINE+PROF+NLP (*r* ≤.7). One typical example is Aomori of SEASON 2012 and SEASON 2013. The graph of Aomori in Multimedia Appendix 2 shows that TRAP+NLP was able to detect a high level of continuous epidemic in SEASON 2013, indicating the effectiveness of the TRAP model. However, as described previously, sometimes it was unable to detect tweets after an epidemic. This remains a subject of future work.

Although the TRAP model achieved higher performance than BASELINE+PROF+NLP, the performance was of a medium level (.4<r≤.7) in Niigata (AREA15), Fukui (AREA 20), Tochigi (AREA 9), Mie (AREA24), Iwate (AREA 3), Kagoshima (AREA 46), and 10 other areas. For example, the graph of Fukui in Multimedia Appendix 2 shows that TRAP+NLP was unable to detect the sequential influenza epidemics in SEASON 2012. There were gaps in other SEASONs. Therefore, the average performance through all SEASONs was medium. TRAP model exhibited poorer performance than BASELINE+PROF+NLP in SEASON 2013 in only one (Kumamoto [AREA 43]) area (see Kumamoto in Multimedia Appendix 2). One of the reasons is medical treatment failure in Kumamoto in the SEASON. That was domestic news, but tons of news on the failure appeared in Twitter stream, causing the bias.

The results show the strong advantages of TRAP+NLP in high-population areas. More importantly, TRAP+NLP never shows worse performance, except in one area. These findings are expected to contribute to similar SNS-based surveillance.

### Parameter Optimization

An important issue was the optimization of parameters used in the model. TRAP model required 5 parameters, *w_GPS_*, *w_PROF_*, *w_IND_*, *w_USERS_*, and *w_TRAP_*, as shown in the equations 1 and 2. As for the 2 parameters *w_GPS_* and *w_PROF_*, we set to 1.0, as comparative models, BASELINE and BASELINE+PROF, set the same weightings. Accordingly, we also set *w_IND_* to 1.0, so the choice of these weightings would be reasonable.

We optimized the other 2 parameters, *w_USERS_* and *w_TRAP_*, in preliminary experiments. We observed changes in the correlation coefficients of high-population areas (top 10) and low-population areas (top 10) by adding 0.01 to the parameter value *w_USERS_* from 0 to 1.0. As a result, 80% of areas (16/20) were found to have a high correlation (*r*>.7) when *w_USERS_* was 0.05 and more. The observation for the parameter *w_TRAP_* was conducted in the same way. Specifically, we tested by adding 1.0 to the parameter value *w_TRAP_* from 0 to 3.0. Consequently, we set *w_USERS_* and *w_TRAP_* to 0.1 and 2.0, respectively, so that this pair could achieve the best performance.

### Limitations and Future Direction

The proposed method has several limitations. First of all, we have methodological limitations when crawling Twitter data and detecting tweet location. Our Twitter crawling method relies on a specific keyword *I-N-FU-RU* (*flu* in Japanese). Further research should crawl all tweets of each person so that we can conduct more detailed analyses, including moving trajectory analysis of a person, a recovery process analysis, and so on. Furthermore, this study handles only the location name as indirect information, but various expressions have been used in indirect messages. Therefore, it would be required to apply location estimation techniques for improving the accuracy of this model.

We also have limitations to use self-reported data by social media users. Generally, social media users are biased toward young- to middle-aged demographics so that their data may not represent the population of interest. In addition, social media data are influenced by a variety of user-dependent factors and surroundings. Thus, this study focused on propagated information about the flu and attempted to embed the sensitivity of social sensors in each stage during epidemics of the flu into a model. However, the sensitivity of social sensors can be affected by multiple factors. For example, if a severe case or death case was reported in a particular subgroup of the population, this event would affect and resensitize trapped sensors. Although this study assumed a straightforward case that a trapped sensor had never been resensitized in a season, there is room for considering relations between the (re)sensitivity of social sensors and the gravity of events.

**Table 4:** Area resolution of surveillance.

Location	Target (number of areas)	Data size (million tweets)
Aramaki [15]	Japan (1)	300
Achrekar [26]	United States (10)	1.9^a^
Culotta [27]	United States (1)	0.5
Kanouch [28]	Japan (1)	300
De Quincy [29]	Europe (1)	0.14
Doan [30]	United States (1)	24^a^
Szomszor [31]	Europe (1)	3

^a^Indicates the number of users in millions.

To improve the detection performance for disease epidemics, it is important to implement functions that enable consideration of various effects related to geographic relations among areas: adjacency (neighborhood or not), accessibility (easy to access or not), and isolation (island or not). Furthermore, this study was conducted to elucidate the current situation of disease epidemics. To predict the spread of disease, we need to develop a method through integration with various prediction models. This would enable us to identify outbreaks of infectious diseases with high accuracy before a wider outbreak.

### Comparison With Prior Work

#### Social Sensors for Health-Related Events

Social media are used to detect various events, such as earthquakes [2-4], political elections [5-7], and stock prices in a market [8]. Among the various applications, the study on health-related event detection referred to as *infodemiology* [1,34] has been gaining much attention from researchers in areas such as air pollution [49], Web-based doctor reviews [50], West Nile virus [9], cholera [10], *Escherichia coli* outbreak [11], dengue fever outbreak [12], and influenza [1,13-33]. One review of the literature reported that half of the SNS-based surveillances are related to influenza (15 of 33 papers) [25]. That is true because influenza is a major worldwide public health concern. In particular, unexpected influenza pandemics, which have been experienced 3 times already in the twentieth century (eg, *Spanish flu*), are global issues.

Twitter is the most frequently used social medium for influenza detection [13-33]. Studies have consistently demonstrated a high correlation between the number of influenza patients and the actual influenza-related tweets. However, most studies targeted only country-level detection. Furthermore, detailed surveillance of areas is rarely conducted, as shown in Table 4. One reason is the volume shortage of tweets in small areas. Therefore, it remains unknown whether a small rural area can achieve the same high performance. One advantage of this study is its investigation performance in areas with small populations.

#### Location Estimation

Location estimation including estimation of the place of residence of someone is an important issue in this study. Although the simplest and most reliable method is to use GPS information, many difficulties can arise. For instance, many users turn off this functionality to maintain the privacy of their information. As a result, location estimation from the SNS original text is necessary. Related studies identified 2 difficulties in location estimation of SNS texts: detecting a location name in tweet messages and disambiguating the location names.

To address these challenges, a collection of location names is necessary. Usually, Wikipedia is used as the basis of a location name dictionary. We also used a location name dictionary obtained from Japanese Wikipedia. As for the location name disambiguation, several methods have been studied [51]. Location-indicative words from tweet data are found by calculating the information gain ratios. Earlier research effort shows that words improve the user location estimation performance. They concluded that the procedure requires little memory: it is fast. Moreover, lexicographers can use it to extract location-indicative words. A probabilistic framework was developed to quantify the spatial variation manifested in search queries [52], which brings them to spatial probabilistic distribution models. One study [53] estimated geographic regions from unstructured, nongeo-referenced text by computing a probability distribution over the surface of the Earth. Another study [54] estimated a city-level user location based purely on the content of tweets, which might include reply tweet information, without the use of any external information, such as a gazetteer or internet protocol (IP) information. Two unsupervised methods [55] have been proposed based on notions of nonlocalness and geometric localness to prune noisy data from tweets. One report [56] described language models of locations using coordinates extracted from geotagged Twitter data. Although this study used geocoding services provided by Google, incorporating such techniques can support future studies.

### Conclusions

This paper proposed a novel approach that uses not only direct information but also indirect information that mentions other places for disease epidemic prediction. We assumed a model by which the indirect information inhibits direct information. In the experiments performed for high-resolution areas (prefecture level), the proposed approach exhibited improved detection performance not only in rural cities but also in urban cities, which demonstrated the effectiveness of the proposed method consisting of a TRAP model and NLP classification.

This model offers sufficient room for additional study. For example, although this study handles only location name as indirect information, various expressions have been used in indirect messages. Therefore, applying location estimation techniques could improve the accuracy of this model. Another limitation of this study is the Twitter crawling method that relies on a specific keyword *I-N-FU-RU*. This method cannot allow the collection of a timeline of tweets of a person. If we crawled all tweets of each person, it could conduct more detailed analyses, including moving trajectory analysis of a person, a recovery process analysis, and so on.

Future work will study worldwide influenza surveillance. Furthermore, we plan to apply this method to other epidemic surveillances and to establish a novel method by integrating various models to exploit their prediction accuracy.

## Appendix

Multimedia Appendix 1 Models used for data aggregation. Note that NLP is the positive/negative classifier, GPS is GPS information, PROF is profile information, and IND is indirect information.

Multimedia Appendix 2 Temporal changes of positive influenza tweets for 3 SEASONs in 6 prefectures in Japan. The x-axis shows the date from the beginning of SEASON 2012 to the end of SEASON 2014, whereas the y-axis shows the tweet ratio and the patient ratio (normalized by the max value in each season). The red line shows the timeline of direct information and indirect information that are aggregated by the proposed model (TRAP+NLP), and the red area shows gold standard timeline. The black dotted line shows the timeline of direct information (BASELINE+PROF+NLP). The blue line shows the timeline of direct information and indirect information that are aggregated in a naive way (LINEAR+NLP). PROF: profile information; NLP: natural language processing.

Multimedia Appendix 3 Relation of the number of tweets (blue bar) and correlation coefficient of TRAP+NLP (red line) and BASELINE+PROF+NLP (dotted black line) for each area. Areas are ordered by populations based on the number of tweets. The x-axis shows the area; the y-axis indicates the correlation coefficient (left side) and the number of tweets (right side). In most areas, the proposed approach (TRAP+NLP) shows a higher correlation ratio than the conventional system. PROF: profile information; NLP: natural language processing.

## Abbreviations

API: application programming interface
GPS: global positioning system
NLP: natural language processing
PROF: profile information
SNS: social networking service
