# Supplementary figures and images for: Twitter-Based Influenza Detection After Flu Peak via Tweets With Indirect Information: Text Mining Study
Source: JMIR Public Health Surveill. 2018 Sep 25;4(3):e65. doi: 10.2196/publichealth.8627 (PMC6231889; doi:10.2196/publichealth.8627)

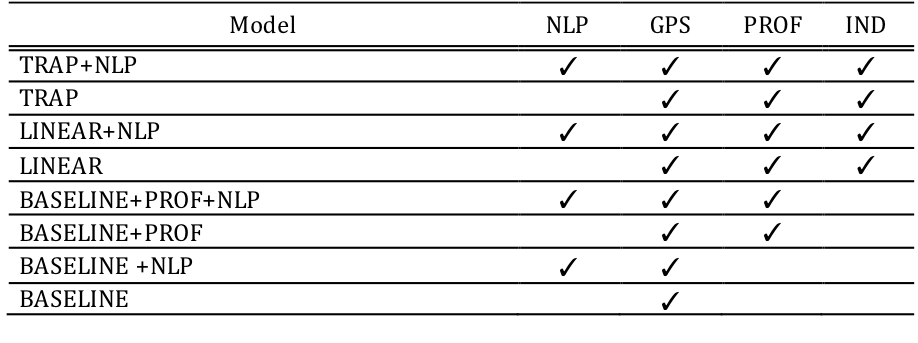

Supplement: Multimedia Appendix 1 [file publichealth_v4i3e65_app1.png]

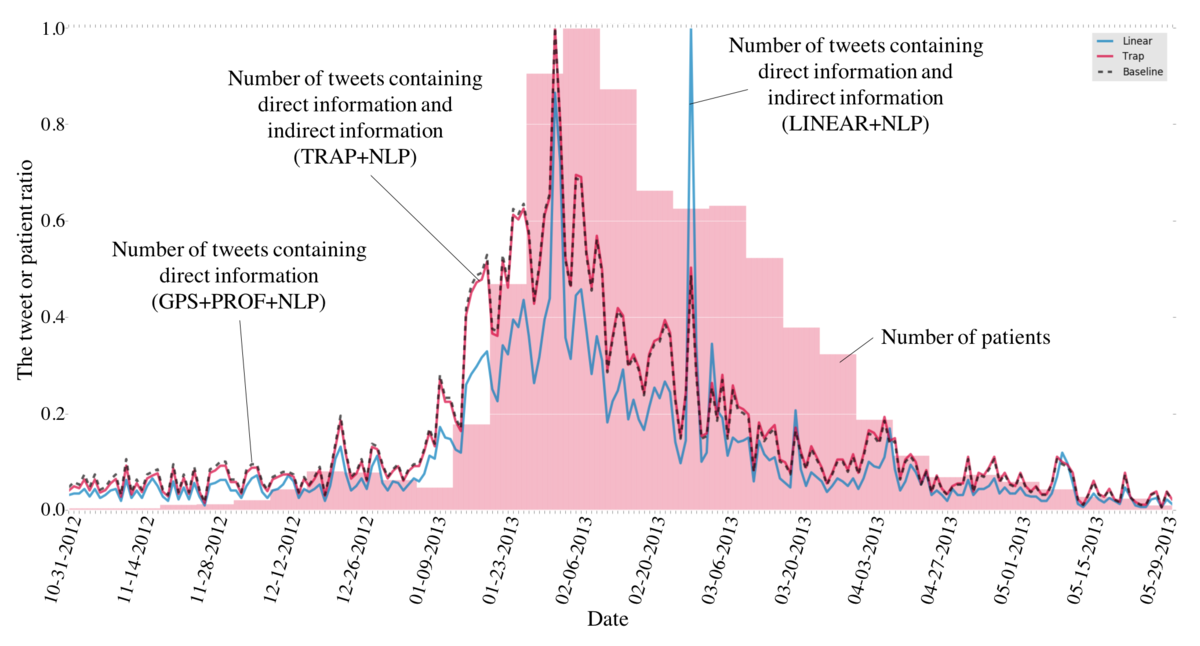

Supplement: Multimedia Appendix 2 [file publichealth_v4i3e65_app2.png]

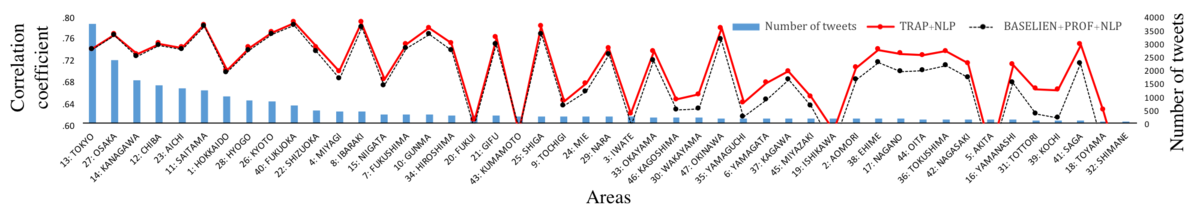

Supplement: Multimedia Appendix 3 [file publichealth_v4i3e65_app3.png]
